# Failed TAVI in TAVI Implantation: TAVI Dislocation Followed by Ensuing Surgical Graft Resection

**DOI:** 10.1155/2017/5086586

**Published:** 2017-04-30

**Authors:** Róbert Novotný, Jaroslav Hlubocký, Tomáš Kovárník, Petr Mitáš, Zuzana Hlubocka, Jan Rulíšek, Sevim Ismihan Gulmez, Shubjiwan Kaur Ghotra, Jaroslav Lindner

**Affiliations:** ^1^2nd Surgical Clinic of Cardiovascular Surgery, General Teaching Hospital, Prague, Czech Republic; ^2^1st Faculty of Medicine, Charles University, Prague, Czech Republic; ^3^2nd Department of Internal Medicine, Department of Cardiology and Angiology, General Teaching Hospital, Prague, Czech Republic; ^4^Clinic of Anesthesiology, Resuscitation and Intensive Medicine, General Teaching Hospital, Prague, Czech Republic

## Abstract

We are presenting a case report of failed valve-in-valve treatment of severe aortic stenosis. A control ultrasonography after TAVI implantation revealed a severe aortic regurgitation of the graft which was subsequently unresolved with postimplantation dilatation. Second TAVI was implanted with cranial dislocation to the aortic root. Patient underwent a CT examination to clarify the TAVI in TAVI position. Patient underwent a surgical resection of TAVI with implantation of biological aortic valve prosthesis. In situations where TAVI treatment fails or is complicated beyond the possibility of endovascular repair, surgical intervention despite its higher risks is the preferred choice.

## 1. Introduction

Transcatheter aortic valve implantation (TAVI) is rapidly progressing in becoming a leading treatment for severe aortic stenosis in high-risk or inoperable patients [1]. Over the past years, we had seen an increase in the number of implanted TAVIs, as well as broadening the spectrum for their medical indications [2]. Implanting TAVI in lower risk patients is one of the major transitions that was observed in recent years [3]. Degeneration of biological valves is extensively documented with median of ten years after surgical replacement [4, 5]. Despite the possibility of a potential failure, these valves are implanted into the elderly patients [5]. On the other hand, the degeneration of TAVI has been documented very rarely [6–8]. Currently, a limited number of works are concerned with systematic reviews of TAVI failures and complications [9, 10]. Dislocation and paravalvular aortic regurgitation are two of the most common technical complications associated with TAVI which must be addressed through endovascular or by surgical means [9, 11, 12].

We are presenting a case report of failed TAVI in TAVI followed by surgical resection and implantation of bioprosthetic aortic valve.

## 2. Case Presentation

77-year-old polymorbid patient with severe aortic stenosis (AVAi: 0,42 cm^2^/m^2^), New York Heart Association (NYHA) Functional Classification: Class II, was admitted into our center for transcatheter aortic valve implantation (TAVI). The patient's past medical history included ischemic heart disease (implantation of Resolute Integrity 3,5 × 12 mm stent into anterior interventricular branch of left coronary artery in June 2015) and transient ischemic attack (TIA) after embolization into right middle cerebral artery (MCA) in February 2014 with reoccurrence in July 2014. The reoccurred TIA after embolization into the right MCA left patient in state of poor mobility with severe neurologic deficit on the left upper and lower extremity. Further patient's past medical history contained arterial hypertension (WHO classification: II), left parailiac lymphadenopathy of unknown origin (based on CT examination in June 2015), and burn trauma to the lower extremities with defects on the lower legs.

Patients Euroscore II (8,95%) with combination of reoccurred TIA after embolization into the MCA, poor mobility with severe residual neurologic deficit, and defect on the lower legs after burn trauma made us classify patient as a “high-risk”; thus, TAVI was indicated as a suitable treatment option.

Under general anaesthesia, through right common femoral artery, a CoreValve 31 mm (Medtronic, Minneapolis, MN, USA) was implanted in a standard manner (through a 6Fr sheath using a guiding wire Confida (Medtronic, Minneapolis, MN, USA), a Nucleus 25 mm (NuMED, Canada) balloon was used to dilate the aortic valve before valve implantation). After the valve implantation was completed, an angiographic and ultrasonographic verification of the valve's position revealed its malposition deep inside the left ventricle. This was the cause of a severe residual aortic regurgitation persisting even after redilation of the valve with NuMED 28 mm (NuMED, Canada) balloon. For this reason, a second CoreValve 31 mm (Medtronic, Minneapolis, MN) was implanted. After implantation, the second graft was dislocated cranially into the aortic root, again with severe residual aortic regurgitation. At the time, due to the increase of aortic diastolic pressure up to 45 mmHg a further intervention was contraindicated. Femoral artery access point was treated with two Angioseals (St. Jude Medical, Angio-Seal™).

**Figure 1:**
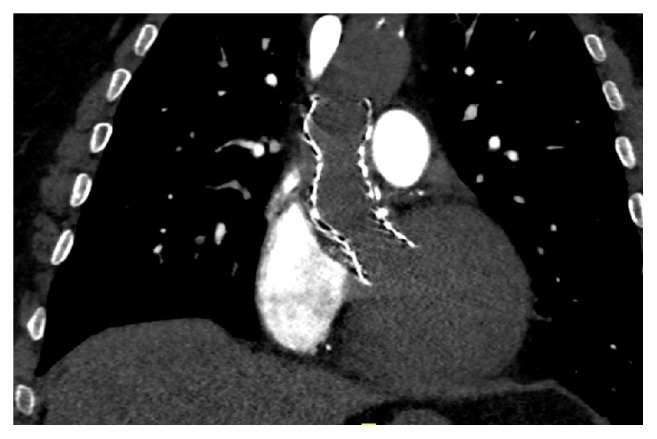
Computed tomography showing positions of TAVI in TAVI.

After recovery from the endovascular procedure marked by multiple complications, a computed tomography (CT) and heart ultrasonography were performed to verify the grafts' positions and the severity of aortic regurgitation causing congestive heart failure of the patient (Figure 1). Following the CT and heart ultrasonography which confirmed a severe residual aortic regurgitation, the patient was indicated for surgical resection of both failed TAVI devices and an aortic valve replacement.

**Figure 2:**
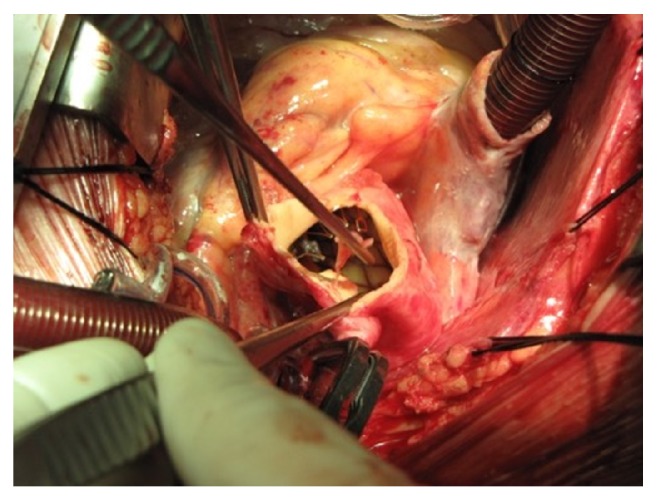
Aortotomy revealing TAVI in aortic root.

The procedure was performed in general anaesthesia, accessing the heart trough complete sternotomy. After standard cannulation for extracorporeal circulation, ascending aorta was cross-clamped. Aortotomy was performed revealing the failed TAVI (Figure 2). Both TAVIs were safely resected and removed from the aortic root and the ascending aorta after they were covered by a frozen saline solution. This caused the metallic structure of TAVIs to contract, thus allowing for an easy removal of the grafts. The aortic annulus was sized after the aortic valve leaflets were resected following the CE Perimount 27 mm valve (Carpentier-Edwards Perimount Magna Ease Aortic Heart Valve) implantation in a standard manner. After aortotomy closure, an ultrasonographic verification of the biological valve function was performed followed by a decannulation and sternotomy closure in a standard manner, respectively.

Patient recovered from the procedure without any further complications and was discharged on the 9th postoperative day with normally functioning aortic biological valve prosthesis.

## 3. Discussion

Conventional aortic valve replacement is the gold standard for the treatment of aortic valve stenosis. However, TAVI has emerged and proved itself as a credible alternative for high-risk patients and patients in advanced age [1, 13]. The latest meta-analysis had shown the safety and efficacy of TAVI when compared to the standard surgical treatment [14]. The other decisive and promising medical indication is an implantation of TAVI into a failing bioprosthetic aortic valve [15]. With more frequent implantation of TAVI in younger and high-risk patients, postprocedural complications such as bleeding, stroke, atrial fibrillation, bacterial endocarditis, late TAVI embolization, TAVI thrombosis, and structural TAVI failure are becoming examined and documented more frequently [9, 16]. On the other hand, periprocedural complications except for paravalvular leaks are documented very rarely [9, 10, 17]. Paravalvular leaks were described as one of the greatest weaknesses of TAVI as they negatively impact mid- and long-term results [18]. However, our presented case shows that even “high-risk” patients may not necessarily benefit from TAVI implantation as the device failure will force the patients to undergo a conventional surgical aortic valve replacement with additional risk of adding TAVI explanation into the equation, thus prolonging and complicating the surgery [19].

TAVI dislocation is a rare but serious complication which if left untreated can have a severe impact on patient's prognosis. Ussia et al. described three main reasons for TAVI dislocation; accidental dislocation immediately after valve implantation, dislocation during the snaring manoeuvre to reposition a low deployment, and intentional dislocation performed as a bailout in cases of coronary ostia impairment [20]. Zahn et al. reported TAVI migration, to the ascending and descending aorta, occurring during few cardiac cycles following the valve deployment [21]. Mechanism of TAVI dislocation and its subsequent placement correction can be greatly affected by the TAVI deployment mechanism: self-expanding versus balloon-expanding [19].

In this presented case, we experienced two types of acute TAVI dislocation. The first type of dislocation was caudal to the left ventricle. This type of dislocation was caused by known tendency of CoreValve (Medtronic, Minneapolis, MN, USA) to move downward due to very high radial forces that are inserted during implantation. The second dislocation was cranial to the aortic root. The main reason for this type of dislocation was postdilation in borderline high position most likely combined with extrasystole during fast pacing.

High efficacy and safety of TAVI procedures in cardiac centers are strongly correlated with high-volume implantation and thorough analysis and documentation of complications and their solutions [22].

## 4. Conclusion

Medical reports documenting TAVI dislocations are very rare. Their true incidence is most likely to be significantly higher. TAVI implantation is associated with a significant learning curve. Understanding the mechanisms of TAVI failures is crucial as the number of implanted TAVIs is rapidly growing.

Submerging CoreValve into ice-cold saline solution causes the shrinkage of the metallic valve skeleton, thus allowing a technically easier resection of the graft assuming that it is not ingrown into the aorta or aortic annulus.
